# Influence of the Support Nature of Copper Catalysts on Catalytic Properties in the Hydrogenation of Fatty Acid Esters

**DOI:** 10.3390/ijms26073289

**Published:** 2025-04-02

**Authors:** Anastasiya Shesterkina, Anna Strekalova, Mikhail Mashkin, Igor Mishin, Kseniia Vikanova, Obid Tursunov, Sergey Dunaev, Alexander Kustov

**Affiliations:** 1 Chemistry Department, Lomonosov Moscow State University, Leninskie Gory 1/3, Moscow 119991, Russia; anastasiia.strelkova@mail.ru (A.S.); mashkin5482@mail.ru (M.M.); ks.vikanova@gmail.com (K.V.); sfdunaev@mail.ru (S.D.); 2 Zelinsky Institute of Organic Chemistry, Russian Academy of Sciences, Leninsky Prospekt 47, Moscow 119991, Russia; anna.strelkova1994@mail.ru (A.S.); igormish42@gmail.com (I.M.); 3 Department of Power Supply and Renewable Energy Sources, National Research University TIIAME, 39 Kari Niyazov, Tashkent 100000, Uzbekistan; obidtursunov@gmail.com

**Keywords:** fatty acid esters, ester hydrogenation, copper catalyst, supported catalyst, methyl butyrate, methyl hexanoate, methyl stearate

## Abstract

Copper-containing catalysts supported on different commercial oxide supports (SiO_2_, Al_2_O_3_, and mixed oxide supports) were prepared by the incipient wetness impregnation method and investigated for the selective hydrogenation of methyl esters (methyl butyrate, methyl hexanoate, methyl stearate) to fatty alcohols. Characterization techniques, including transmission (TEM) and scanning electron microscopy (SEM), X-ray diffraction (XRD), N_2_ adsorption–desorption isotherms, and the temperature-programmed hydrogen reduction (H_2_-TPR) method, were utilized and revealed the relationship between catalyst properties and its structure. The best results of catalytic activity were obtained in the presence of the Cu catalyst supported on SiO_2_ with co-precipitated Al_2_O_3_, where the conversion of esters was above 50% with a selectivity for the corresponding alcohols of 40–70%. This efficient and inexpensive Cu-based catalyst can be widely used in industrial production, which is conducive to promoting the development of non-precious metal catalysts in the biomass industry.

## 1. Introduction

Fatty alcohols (FAs) are highly important base chemicals widely used in a variety of chemical industry areas, including the production of plastics, surfactants, lubricants, cosmetics, and many others [1,2,3,4]. Globally, the market for fatty alcohols already reached USD 6 billion in 2022 and will continue to grow [5,6]. In order to fulfill this demand, two pathways can be used for the commercial production of fatty acids: synthetic, which implies ethylene oligomerization in the Ziegler process [7], or biobased, via the selective catalytic hydrogenation of fatty acid methyl esters (FAMEs) obtained from vegetable oils [8,9]. Undoubtedly, the second approach seems more promising and sustainable due to the use of renewable feedstocks, but from an economic point of view, there is a large room for improvement, and one of the most important factors affecting FA production is the choice of the suitable catalyst [9].

Traditionally, Cu-Cr-based catalysts (Adkins catalyst), which are effective only at high reaction temperatures of 200–400 °C and a hydrogen pressure of 10–20 MPa, have been applied to produce fatty alcohols from fatty acid/natural oil hydrogenation [10,11,12]. However, given the potential environmental issues caused by Cr^6+^ leaching and the severe reaction conditions, researchers face the challenge of replacing these systems with more efficient and non-toxic catalysts. Heterogeneous catalysts for this process are represented by both noble (Rh, Pd, Ru, Pt, Re) [13,14,15,16,17] and non-noble metals (Cu, Ni, Co) [18,19,20,21]. The main drawbacks of noble metal-based catalysts are high price and low selectivity, which are due to high metal reactivity and the fast formation of by-products in the reaction mixture [2].

From this perspective, the catalyst for FAME hydrogenation toward FAs should be selective in order to ensure a high yield of the targeted products active at relatively mild conditions in order to decrease energy costs, be non-toxic, and not contain noble metals. Catalysts based on the Co and Ni ones possess high ester hydrogenation activity and resistance to sintering. Unfortunately, they are also very active for decarbonylation/decarboxylation and C-C bond hydrogenolysis, which is undesirable because of the reduced yield of alcohols. The Cr-free copper catalysts, in turn, receive attention due to their high selectivity to fatty alcohols, but they are active at high temperatures (above 200 °C), which leads to their rapid deactivation due to particle sintering. This can be overcome by the modification with a second metal (such as Sn, Fe, Re, and In) or the interaction with the supports [22,23,24,25]. Studies of copper catalysts based on silica have shown that both the high dispersion of copper species and the strong interaction between the metal and the support are important factors for achieving high activity and stability during the hydrogenation of esters to alcohols. This is similar to the behavior of the Cu-Cr catalytic system [26,27]. He et al. [18] studied an optimized CuO/ZnO/Al_2_O_3_ composite catalyst providing a 98% yield of stearyl alcohol in the hydrogenation of ethyl stearate at 230 °C for 4 h. It has been shown that the high catalytic performance was attributed to relatively high dispersion (small crystal size) and the high reducibility of copper species because of the strong electronic interaction among the components of the system: CuO, ZnO, and Al_2_O_3_.

It is shown that the simultaneous presence of Cu^0^ and Cu^+^ centers on the surface of the catalyst has a predominant effect on the efficiency of ester hydrogenation [28,29]. It has been demonstrated recently that the Cu^0^ species in the reduced Cu/SiO_2_ catalyst can be oxidized to Cu^+^ by ethyl acetate and, at the same time, Cu^+^ ions can also be reduced to Cu^0^ under hydrogenation reaction conditions. Thus, we can conclude that the repeated cycle between Cu^0^ and Cu^+^ exists during the hydrogenation of esters.

To obtain highly dispersed and stabilized Cu nanoparticles, a strong metal support interaction or sophisticated preparation is required. However, supported Cu over typical reducible metal oxides, such as TiO_2_ and CeO_2_, shows a dramatically low activity due to the electron deficiency of the Cu surface induced by those reducible metal oxides [30,31,32].

Alternatively, copper phyllosilicate offers high Cu dispersion (>65%) over non-reactive support. This could be attributed to the strong interaction between Cu-SiO_2_ in the octahedral sites of chrysocolla structures, which leads to the formation of Cu^+^ species retained in the catalysts [33,34]. In fact, the presence of Cu^+^ species enhanced Cu dispersion, presumably preventing Cu agglomeration, which further increased the activity.

**Figure 1:**
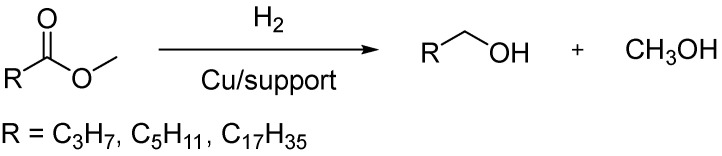
The general scheme of the reaction of hydrogenation of esters into alcohols considered in this work.

The aim of this work is to compare the efficiencies of copper catalysts supported on different oxides (γ-Al_2_O_3_, SiO_2_-Al_2_O_3_, Al_2_O_3_-SiO_2_, and SiO_2_) into the liquid-phase hydrogenation of model fatty acid esters (methyl butyrate, methyl hexanoate, methyl stearate) to the appropriate fatty alcohols (Figure 1). Various physico-chemical methods (H_2_-TPR, TEM, SEM, XRD, and N_2_ physisorption) are used to thoroughly characterize the catalysts. The influence of the support nature, its textural properties, and the composition of the catalyst on the catalyst’s efficiency are analyzed.

## 2. Results

### 2.1. Physico-Chemical Properties of Catalysts

**Figure 2:**
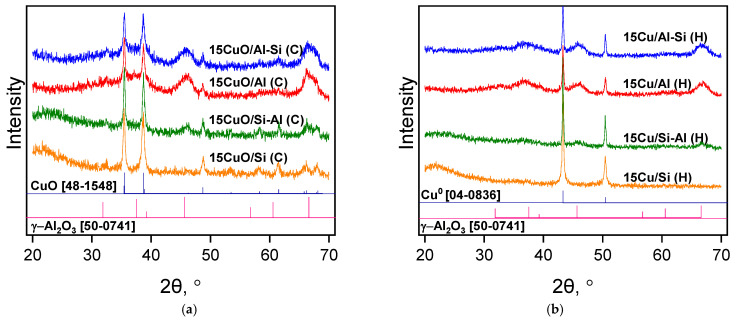
XRD patterns of the calcined (**a**) and reduced (**b**) copper catalysts on different supports.

The XRD method was used to study the phase composition of calcined (C) and reduced (H) catalysts with 15 wt.% Cu and determine the average size particle of crystallites (Figure 2). The silica support exhibits no diffraction, indicating that it is entirely amorphous. For the samples supported on Al_2_O_3_-containing supports, main diffraction lines are present at 2θ values of 37.5°, 45.8°, and 67.2°, which can be attributed to the γ-Al_2_O_3_ structure according to the JCPDS card No. 50–0741. The X-ray profile of the calcined samples supported on all supports shows two main reflections at 2θ = 35.6° and 38.7° and low-intensity reflections at 48.4° and 62.1°, which correspond to the characteristic diffraction lines of crystalline CuO, in agreement with JCPDS card No. 48-1548 [35]. Typical diffraction peaks of Cu^0^ at 2θ = 43.3° and 50.4° were observed in the XRD profiles of all reduced samples, which were attributed to the (111) and (200) faces of Cu (JCPDS No. 04–0836), respectively. The highest intensity of the diffraction peaks of Cu^0^ was observed for a sample supported on Si-Al, which indicates the formation of large Cu particles with high crystallinity.

The particle sizes CuO and Cu^0^ calculated by Scherrer’s formula for calcined and reduced catalysts, respectively, are presented in Table 1. The crystallite size of copper oxide in calcined samples at 400 °C for 3 h is about 22 nm. The reduction in calcined samples in hydrogen at 350 °C leads to an increase in the size of the crystallites. However, the nature of the support and its textural properties affect the resulting crystallite size of metallic copper particles. The support on the Si-Al carrier led to the formation of large particles of copper with a size of 42 nm.

The actual amount of Cu in catalysts after impregnation was measured by the ICP-AES method, and the results are provided in Table 2. Despite the fact that the method of applying copper to different supports was identical and the mass content of copper during application was 15 wt.%, the ICP results differ for all samples and are somewhat less than the theoretically set value during deposition.

**Figure 3:**
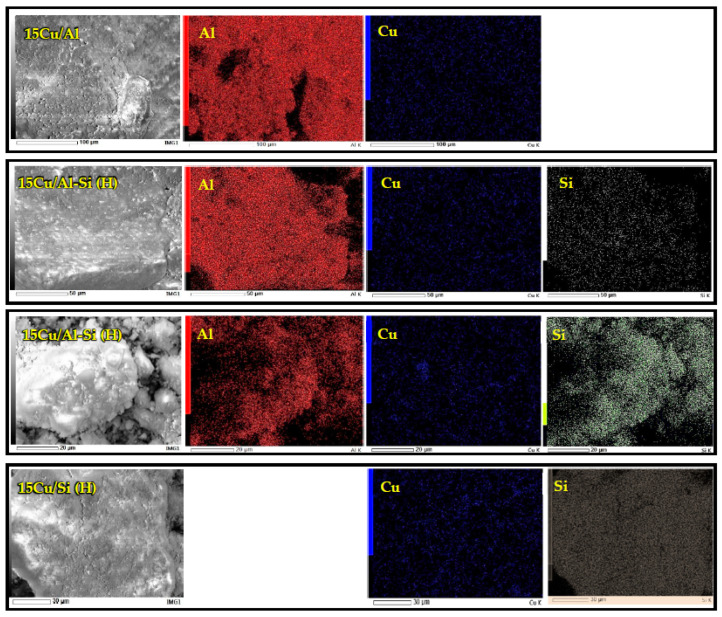
SEM-EDX element mapping of reduced (H) copper catalysts with 15 wt.% on different supports.

SEM-EDX analysis of reduced (H) catalysts was carried out to examine the distribution of supported metals on the surface of the supports. Figure 3 demonstrates the SEM-EDX elemental mapping of the supported catalysts. The obtained images show that Cu (blue) particles were finely dispersed over the entire surface of all supports (gray background). However, local areas of the formation of copper particle agglomerates can be seen in the 15Cu/Si-Al(H) sample.

The energy dispersion analysis (Table 3) gave an understanding of the obtained mass content of copper on the outer surface of the catalysts. It is worth noting that a significant deviation of the experimentally obtained mass contents of copper from the theoretically set ratio and ICP-AES data was observed in all supported catalysts. The reason for this is probably that the deposition of copper occurs mainly on the inner surface of the carrier, which is not available for analysis by the SEM-EDX method. However, most of the available metal on the outer surface of the support, using the same method of sample synthesis, was found on a sample supported on a Si-Al support.

**Figure 4:**
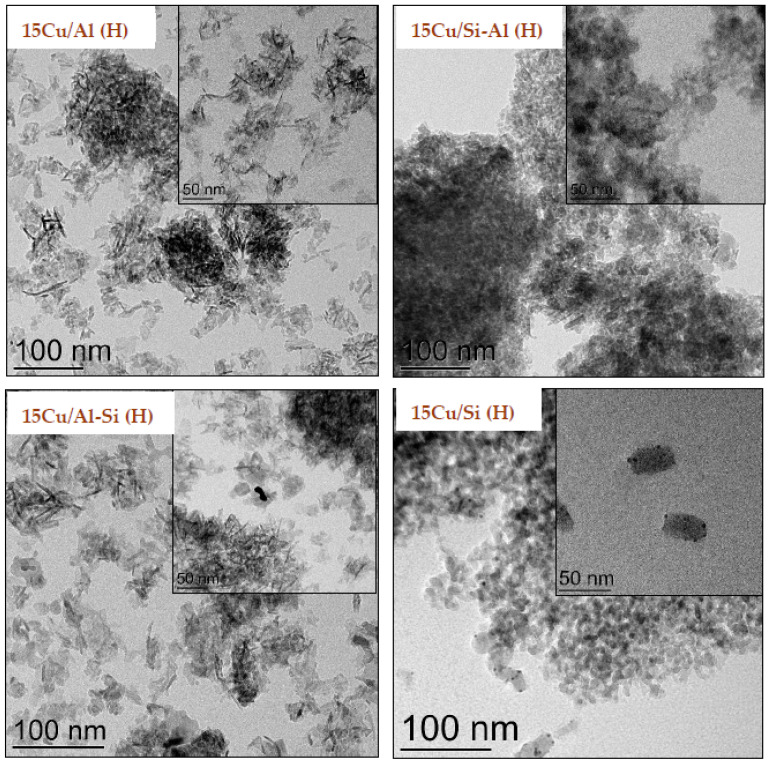
TEM images of reduced 15Cu (H) catalysts supported on different supports.

To further investigate the effect of the support on metal dispersion, the reduced (H) samples were subsequently analyzed using transmission electron microscopy (TEM), as shown in Figure 4. Micrographs of catalysts on the Al_2_O_3_-containing support (15Cu/Al (H) and 15Cu/Al-Si (H)) are represented by a typical microstructure characterizing the needle-like nature of the carrier, on which a highly dispersed phase of spherical nanoparticles with an average Cu particle size of ~2.7 nm can be seen, as well as aggregated particles of about 20 nm, which is consistent with the XRD data of these samples. Cu^0^ particles in reduced 15Cu/Si-Al and 15Cu/Si catalysts were well dispersed on the surface of the support with an average particle size of 1.7 and 1.9 nm, respectively.

The porosity of the initial supports and synthesized catalysts was investigated by the N_2_ adsorption method (Table 4). All supports had similar characteristics described by the suppliers. It should be noted that the addition of a copper precursor can lead to a change in the physical properties of the materials. It is important to note that in all cases the microporosity of both carriers and catalysts is extremely low, so the contribution of micropores to the total volume is insignificant. In the case of Al_2_O_3_ support, the increase in the surface area together with the increase in pore volume was observed after impregnation by the copper solution that could be caused by the creation of additional defects due to interaction between the precursor and the carrier. For the catalysts based on SiO_2_, SiO_2_-Al_2_O_3_, and Al_2_O_3_-SiO_2_, the addition of a metallic phase led to a decrease in total pore volume and surface area. The increase in average pore volume might be caused by the affection of acidic copper precursor solution on the pore walls, leading to the partial destruction of smaller pores and the formation of larger ones [36]. On the other hand, additional pore collapse can be followed by calcination after the impregnation step, which also explains the changes in BET area values.

**Figure 5:**
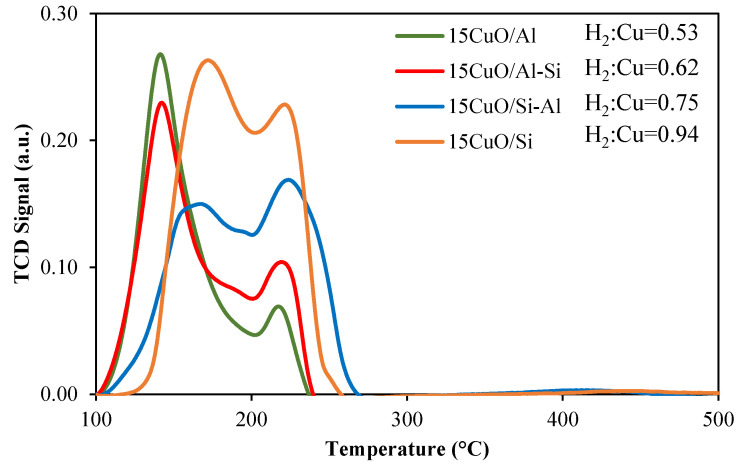
TPR-H_2_ plot of the calcined 15CuO/X (C) catalysts.

The reducibility of the prepared calcined catalysts was studied by the H_2_-TPR method. The TPR profiles of the catalysts are demonstrated in Figure 5. As is noted by Mambetova et al. [37], the typical temperature for the reduction in bulk copper oxide is near 250 °C; however, this peak can be shifted depending on the carrier’s nature and metal dispersion in the case of supported catalysts. On the curves of the samples with high alumina content (15Cu/Al and 15Cu/Al-Si), the main peak is located at 144 °C, referring to the reduction in copper oxide to metallic copper. In our case, the low temperature of the hydrogen consumption peak can be explained by the reduction in the nanoparticles of small sizes on the outer surface of the support. Mazarío et al. [38] observed the reduction peak of finely dispersed copper nanoparticles supported by silica with maxima at 190 °C. The second peak in all samples located at 223 °C can be referred to as the reduction in copper agglomerates [39]. The calculated H_2_:Cu value for the samples 15CuO/Al and 15CuO/Al-Si is close to 0.5, stating that copper reduction is incomplete in these cases. It can be assumed that while the reduction in small nanoparticles on the outer surface of the support occurs at lower temperatures, the reduction in CuO in the narrow pores is hindered.

However, the first reduction peak is shifted toward higher temperatures in the case of catalysts supported by the carriers with high silica content (15Cu/Si and 15Cu/Si-Al). This can be explained by the increase in the sizes of individual particles located on the support surface, which was confirmed during SEM and TEM studies. The position of the second reduction peak stayed the same in silica-enriched samples. It should be noted that in our case, the calcination temperature of 300 °C makes it possible to completely reduce copper oxide to metallic copper, which is confirmed by the XRD method (Figure 2b).

### 2.2. Catalytic Activity

**Figure 6:**
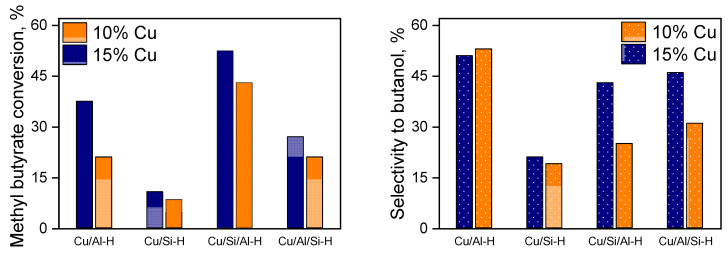
Influence of the support nature of reduced Cu-based catalysts (**left**) on the methyl butyrate conversion (**right**) on the selectivity to butanol at 2.5 MPa H_2_ and 240 °C.

The catalytic properties of reduced copper-containing catalysts were studied in the hydrogenation reaction of methyl esters of fatty acids. The first representative of a number of fatty acid esters—methyl butyrate—was chosen as a model substrate. The catalytic properties of reduced Cu-based catalysts were studied under relatively mild reaction conditions (240 °C and a hydrogen pressure of 2.5–4 MPa). The hydrogenation reaction was carried out in the hexane as a solvent. The target product of the methyl butyrate hydrogenation reaction is butanol; however, butyl butyrate and methanol were also found in the reaction products. Butyl butyrate is formed by transesterification with unreacted methyl butyrate and, depending on the composition of the catalyst, its formation is different. Similar results were obtained and described in the hydrogenation of methyl octanoate in the presence of nickel catalysts [4]. The influence of the loading of copper and the support nature on the catalytic properties was investigated. As can be seen in Figure 6, an increase in the copper content to 15 wt.% in all supported catalysts leads to an increase in both the conversion of methyl butyrate and the increase in butanol selectivity. As can be noted, the nature of the support has an impact on the conversion and selectivity of the process. The catalysts supported on SiO_2_ showed weak catalytic properties, and the methyl butyrate conversion did not exceed 10%.

The use of Al_2_O_3_ increased both the selectivity to 50% and the methyl butyrate conversion to 38%. The best results toward methyl butyrate conversion were obtained in the presence of the Cu catalyst supported on SiO_2_ with co-precipitated 25% Al_2_O_3_ (Si-Al); however, the selectivity of butyl alcohol on this catalyst was lower than on an individual Al support. Probably, the SiO_2_ support affects the course of a side process transesterification to butyl butyrate.

**Figure 7:**
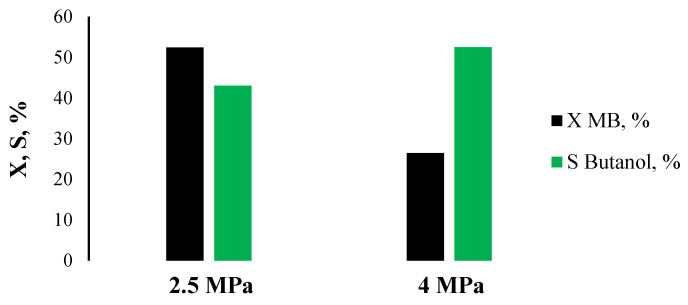
The effect of hydrogen pressure on catalytic properties in the methyl butyrate hydrogenation on the 15Cu/Si-Al(H) catalyst.

**Figure 8:**
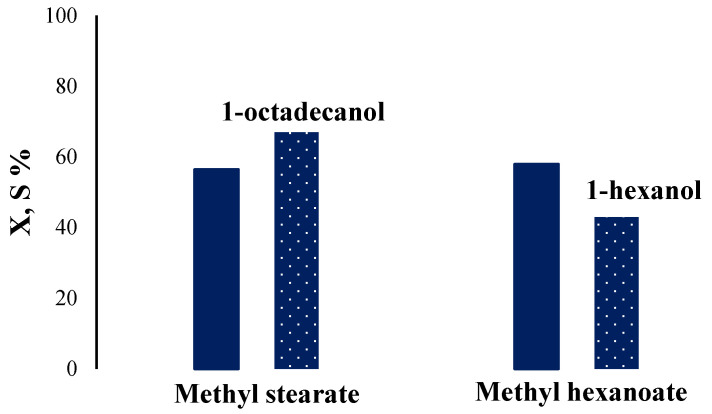
The catalytic properties of the 15Cu/Si-Al(H) catalyst in the methyl stearate and methyl hexanoate hydrogenation.

The work also conducted research on the effect of hydrogen pressure on catalytic properties. As can be seen in Figure 7, an increase in pressure to 4 MPa suppresses the process of ether hydrogenation, while at the same time, the degree of conversion (X) decreased by two times. For this reason, further experiments on the study of catalytic properties were carried out at a hydrogen pressure of 2.5 MPa. The 15Cu/Si-Al catalyst, which showed the best catalytic properties in methyl butyrate hydrogenation, was investigated in the hydrogenation of the methyl stearate and methyl hexanoate (Figure 8). The conversion of esters (X) was above 50% with high selectivity (S) for the target alcohols—1-octadecanol 67% and 1-hexanol 43%.

## 3. Materials and Methods

### 3.1. Materials

Commercial γ-Al_2_O_3_ with S_BET_ 250 m^2^/g and V_por_ = 1.05 cm^3^/g (Saint-Gobain, Courbevoie, France), SiO_2_ with co-precipitated 25% Al_2_O_3_ with S_BET_ 400 m^2^/g and V_por_ = 0.6 cm^3^/g (Saint-Gobain, France), Al_2_O_3_ with co-precipitated 25% SiO_2_ with S_BET_ 225 m^2^/g and V_por_ = 0.7 cm^3^/g (Saint-Gobain, France), and SiO_2_ with S_BET_ 250 m^2^/g and V_por_ = 1.01 cm^3^/g (Saint-Gobain, France) were used as supports. The supports were denoted as Al, Si-Al, Al-Si, and Si, respectively. Prior to the synthesis of catalysts, all supports were subjected to temperature treatment at 300 °C in an air atmosphere to remove impurities and adsorbed water.

Reagents used in this work include copper (II) nitrate trihydrate, Cu(NO_3_)_2_·3H_2_O, supplied by ABCR GmbH (Karlsruhe, Germany). Fresh distilled water was used to prepare the necessary solutions. Methyl butyrate (MB), methyl hexanoate (MH), and methyl stearate (MS) were supplied by Acros Organics (Geel, Belgium).

### 3.2. Catalyst Preparation

Cu-containing catalysts with loadings of 10 and 15% wt. were prepared by incipient wetness impregnation of supports. The required amount of the impregnation solution of Cu(NO_3_)_2_ in distilled water was added dropwise to supports under intensive mixing and left at room temperature for 1 h. After that, the impregnated samples were dried for 12 h at a temperature of 100 °C and calcined at 400 °C for 3 h. The calcined catalysts were denoted as CuO/Al (C), CuO/Si-Al (C), CuO/Al-Si (C), and CuO/Si (C), respectively.

The obtained calcined catalysts were reduced in a hydrogen flow in a glass reactor at a temperature of 350 °C for 2 h. Reduced catalysts were denoted as Cu/Al (H), Cu/Si-Al (H), Cu/Al-Si (H), and Cu/Si (H), respectively.

### 3.3. Catalyst Characterization

The textural properties and surface elemental distribution were evaluated using the scanning electron microscope JEOL JSM-6000PLUS Neoscope II with the energy-dispersive spectrometer JED-2300 (JEOL Ltd., Akishima, Japan) at a 15 kV accelerating voltage.

The morphological properties of the catalysts were investigated by the HRTEM method using a JEOL JEM-2100 transmission electron microscope.

The XRD analysis of the samples was performed using an ARL X’TRA diffractometer (Thermo Fisher Scientific, Waltham, MA, USA) with monochromatic Cu-Kα radiation (40 kV, 40 mA) with a scanning rate of 1.2° per minute in the angular range of 20–70° on the 2θ scale. ICCD data were used for identification purposes.

The character of the reduction in the synthesized catalysts was investigated by the temperature-programmed hydrogen reduction (H_2_-TPR) method using an AutoChem 2950HP instrument (Micromeritics Instrument Corp., Norcross, GA, USA). Before TPR runs, the samples (0.10 ± 0.01 g of the fraction 0.25–0.35 mm) were kept in an Ar flow at 350 °C for 1 h, then they were cooled to 20 °C. Then, the samples were heated from 20 °C to 500 °C with a heating rate of 10 °C min^−1^ in a flow (30 mL min^−1^) of a reducing mixture (5% H_2_ in Ar) and reduced at 500 °C until H_2_ consumption became negligible. The reduced sample was cooled down to room temperature in an Ar flow.

The N_2_ adsorption–desorption isotherms were measured by Micromeritics ASAP 2020. Prior to the acquisition of the adsorption isotherm, the samples were degassed for 3 h at 130 °C and 350 °C under a residual pressure of 0.8 Pa. The BET method was used to calculate the specific surface area of the sample. Pore size distributions for mesopores were determined by the Barrett–Joyner–Halenda (BJH) method. The total pore volume was evaluated at p/po = 0.99. The cumulative volume at desorption in the BJH method was taken as a mesopore volume. The micropore volume was calculated as the difference between the total pore volume and the mesopore volume. The mesopore-specific surface area was calculated as cumulative at desorption in the BJH method. The micropore size distribution was calculated according to the Horwath–Kawazoe model in assumption of the cylinder shape of the pores.

The spectral atomic emission method with inductively coupled plasma (ICP-AES) was used to determine the content of Cu in solutions. The measurements were performed using an iCAP 6300 Radial View Thermo Fisher Scientific Inc. device (Waltham, MA, USA). The stock solutions were diluted 1000-fold with an HCl solution. The standard samples of the Cu solutions were used to calibrate the spectrometer.

### 3.4. Catalytic Activity Test

The catalytic properties of the pre-reduced copper catalysts were investigated in the liquid-phase hydrogenation of fatty acid esters (methyl butyrate (MB) (99%, Acros Organics), methyl hexanoate (MH) (99%, Acros Organics), and methyl stearate (MS) (99%, Acros Organics) with molecular hydrogen using a stainless-steel “Parr” autoclave system (Parr Instrument Company, Moline, IL, USA) (50 mL) with a probe-withdrawing valve. The substrate solution in hexane (0.1 M in 30 mL) as a solvent with dodecane (n_syb_/n_st_ = 1:1) as an internal standard was loaded into an autoclave. The loading of the catalyst was 0.2 g. The autoclave was subsequently purged three times with hydrogen before filling the vessel to the desired pressure. Reactions were carried out under H_2_ pressure of 2.5–4 MPa with the temperature at 240 °C at the stirring rate of 500 rpm and 2 h reaction duration. On completion of the reaction, both heating and stirring were stopped, and the autoclave was cooled in ice-cold water. After cooling the autoclave, the reaction mixture was separated from the catalyst by centrifugation. The product distribution was determined by a Crystal 5000 chromatograph (Chromatek, Yekaterinburg, Russia) using the ZB-5 capillary column (30 m × 0.53 mm × 1.50 μm) equipped with one flame ionization detector with an internal standard method and a column temperature of 220 °C. The volume of the analyzed sample was 1 µL. Argon was used as a carrier gas at a flow rate of 30 mL min^−1^. Calibration was performed using known concentrations of all reactants and products in order to determine the correct response factors. The catalytic properties of the samples were characterized by ester conversion, as well as by the selectivity of alcohol formation. The ratio of the peak areas of the product and the standard served as an analytical parameter in the calculation of the conversion of the ester, as well as selectivity for hydrogenation products. The conversion (*X*) was determined by the ratio of the peak areas of the substrate and the standard.

## 4. Conclusions

The current work investigates the activity of the copper-containing catalysts based on different supports in the reaction of liquid-phase hydrogenation of fatty acid methyl esters under mild reaction conditions (240 °C and 2.5 MPa of H_2_). It has been shown that the catalytic activity of copper catalysts is influenced not only by the nature of the carrier but also by the textural characteristics of the support and the size of the resulting copper particles. The phase composition of the catalysts was studied by a variety of techniques. XRD analysis of the samples allowed us to conclude the presence of CuO and metallic copper phases. In our case, these results seem to be reliable due to the high resolution of the peaks on XRD patterns as well as the high amount of copper in the samples. Further, the phase composition was confirmed by TPR measurements where together with peak positions, the calculated hydrogen-to-copper ratio confirmed the presence of CuO and Cu phases. SEM investigations allowed us to demonstrate the difference in nanoparticle morphologies of different catalysts. The EDX results indeed deviate from the theoretical amount of copper, which in our case might be followed by an uneven distribution of copper through the catalyst nanoparticle with a higher Cu concentration in the bulk and a lower concentration on the nanoparticle surface. So, it was proved that most of the available metal Cu on the outer surface of the support was detected for the 15Cu/Si-Al(H) catalyst.

The best results toward methyl butyrate conversion were obtained in the presence of the Cu catalyst supported on SiO_2_ with co-precipitated 25% Al_2_O_3_ (Si-Al). Also, large copper crystallites were found in the 15Cu/Si-Al(H) catalyst by the XRD method, which is probably responsible for increased catalytic activity. In the presence of the 15Cu/Si-Al(H) catalyst, the conversion of esters above 50% was obtained with a selectivity for the corresponding alcohols of 40–70%.

This efficient and inexpensive catalyst can be widely used in industrial production, which is conducive to promoting the development of non-precious metal catalysts in the biomass industry.

## Figures and Tables

**Table 1:** Average sizes of CuO crystallites in calcined (C) catalysts and Cu^0^ crystallites in reduced (H) catalysts from XRD data.

Catalyst	Average Size of CuO Crystallites, nm	Average Size of Cu^0^ Crystallites, nm
15Cu/Al	22.5	35.6
15Cu/Al-Si	22.5	30
15Cu/Si-Al	22.7	42.4
15Cu/Si	19.8	27.7

**Table 2:** ICP-AES studies of the prepared catalysts.

Sample	Elements Content, %wt
	Cu
15Cu/Al (H)	11.2
15Cu/Al-Si (H)	11.6
15Cu/Si-Al (H)	11.4
15Cu/Si (H)	10.7

**Table 3:** Mass contents of Cu in the supported catalysts obtained by SEM-EDX.

Catalyst	Theoretical Content Cu, wt.%	Received Content Cu, wt.%
15Cu/Al (H)	15	4.1
15Cu/Al-Si (H)	15	4.3
15Cu/Si-Al (H)	15	6.1
15Cu/Si (H)	15	4.5

**Table 4:** N_2_ adsorption studies of the supports and calcined catalysts.

Sample	BET Surface, m^2^/g	V_total_, cm^3^/g	V_micro_, cm^3^/g	Average Pore Size, nm
Al_2_O_3_	254	0.80	0.005	12.5
15CuO/Al (C)	321	1.02	0	12.6
SiO_2_	236	0.92	0	15.5
15CuO/Si (C)	196	0.79	0.006	16.0
SiO_2_-Al_2_O_3_	410	0.60	0.006	5.8
15CuO/Si-Al (C)	291	0.47	0	6.5
Al_2_O_3_-SiO_2_	232	0.72	0.002	12.4
15CuO/Al-Si (C)	189	0.59	0.004	12.5

## Data Availability

The original contributions presented in the study are included in the article, and further inquiries can be directed to the corresponding author/s.
